# Calcium channel blocker amlodipine besylate therapy is associated with reduced case fatality rate of COVID-19 patients with hypertension

**DOI:** 10.1038/s41421-020-00235-0

**Published:** 2020-12-22

**Authors:** Lei-Ke Zhang, Yuan Sun, Haolong Zeng, Qingxing Wang, Xiaming Jiang, Wei-Juan Shang, Yan Wu, Shufen Li, Yu-Lan Zhang, Zhao-Nian Hao, Hongbo Chen, Runming Jin, Wei Liu, Hao Li, Ke Peng, Gengfu Xiao

**Affiliations:** 1 State Key Laboratory of Virology, Wuhan Institute of Virology, Center for Biosafety Mega-Science, Chinese Academy of Sciences, Wuhan, Hubei 430071 China; 2 Department of Laboratory Medicine, Tongji Hospital, Tongji Medical College, Huazhong University of Science and Technology, Wuhan, Hubei 430030 China; 3 Tongji Medical College, Huazhong University of Science and Technology, Wuhan, Hubei 430022 China; 4 Department of Pediatrics, Union Hospital, Tongji Medical College, Huazhong University of Science and Technology, Wuhan, Hubei 430022 China; 5 Beijing Institute of Microbiology and Epidemiology, State Key Laboratory of Pathogen and Biosecurity, Beijing 100071, China

**Keywords:** Mechanisms of disease, Cell biology

## Abstract

The coronavirus disease (COVID-19) caused by the novel severe acute respiratory syndrome coronavirus 2 (SARS-CoV-2) has now spread to >200 countries posing a global public health concern. Patients with comorbidity, such as hypertension suffer more severe infection with elevated mortality. The development of effective antiviral drugs is in urgent need to treat COVID-19 patients. Here, we report that calcium channel blockers (CCBs), a type of antihypertensive drug that is widely used in clinics, inhibited the post-entry replication events of SARS-CoV-2 in vitro, while no in vitro anti-SARS-CoV-2 effect was observed for the two other major types of antihypertensive drugs, namely, angiotensin-converting enzyme inhibitors and angiotensin II receptor blockers. CCB combined with chloroquine showed a significantly enhanced anti-SARS-CoV-2 efficacy. A retrospective clinical investigation on hospitalized COVID-19 patients with hypertension as the only comorbidity revealed that the CCB amlodipine besylate therapy was associated with a decreased case fatality rate. The results from this study suggest that CCB administration to COVID-19 patients with hypertension as the comorbidity might improve the disease outcome.

## Introduction

The emerging coronavirus disease (COVID-19) is caused by an infection of severe acute respiratory syndrome coronavirus 2 (SARS-CoV-2)^1,2^ and has similar symptoms to SARS-CoV, including fever, cough, dyspnea, etc., and can result in multiple organ dysfunction syndrome and, in severe cases, death^3^. SARS-CoV-2 transmission has caused a global pandemic, posing a serious threat to public health. Until 8 October 2020, there were over 36,000,000 confirmed COVID-19 cases with >1,000,000 deaths from SARS-CoV-2 infection around the world. The development of effective antiviral drugs is urgently needed to contain the current pandemic of SARS-CoV-2 and to counteract its potential reemergence in the future.

So far, no antiviral drug for SARS-CoV-2 has been officially proved to be effective in treating COVID-19 patients. Compared with de novo drug development, which normally takes years of development and evaluation, repurposing of preexisting drugs that are in clinical use is one of the quickest strategies for developing drugs against emerging viruses^4^. Our recent study reported that remdesivir, favipiravir, and chloroquine (CQ) had a distinct in vitro anti-SARS-CoV-2 effect^5^. CQ and hydroxychloroquine (HCQ) are antimalarial drugs with safe records in clinical administration^6^. Given its approved status, it was quickly tested in clinics, while a recent observational study indicated that HCQ administration was not associated with either a greatly lowered or an increased risk of the composite end point of death for COVID-19 patients^7^. Remdesivir was first developed for treating Ebola virus^8^, and showed a strong anti-SARS-CoV and MERS-CoV activity^9,10^. A double-blind, randomized, placebo-controlled trial has been initiated to assess the efficacy and safety of remdesivir to treat COVID-19, and the results indicated that remdesivir was superior to placebo in shortening the time to recovery in adults hospitalized with COVID-19 (ref. ^11^). Favipiravir is an anti-influenza drug that has been approved for clinical use in Japan and, very recently, in China. Similar with remdesivir, favipiravir has also been registered in clinical trials to evaluate its efficacy in treating COVID-19 (ref. ^4^), and the results indicated that favipiravir treatment is independently associated with faster viral clearance^12^. These progresses strongly support the endeavor of repurposing approved drugs for COVID-19 treatment.

The most affected COVID-19 patients are the elderly, who often have comorbidities, such as hypertension, diabetes, cardiovascular disease, etc.^13^. These patients suffer more severe infection outcomes, with a significantly higher case fatality rate (CFR)^13^. The current therapeutic regime is largely symptomatic treatment, and specific evaluation of drug treatment for COVID-19 patients with different comorbidities is still lacking. The identification of more drug candidates with anti-SARS-CoV-2 efficacy would help to provide more options from which safe and effective drugs can be selected, and/or combined for personalized medication for the patients on an individual level.

Calcium channel blockers (CCBs) are widely used in the clinics for treating hypertension, angina pectoris, and supraventricular arrhythmias^14^. CCBs were also recently reported to have an antiviral effect against several emerging viruses, including bunyaviruses, arenaviruses, and flaviviruses^15–17^. About 30% of SARS-CoV-2 patients have hypertension as a comorbidity, and these patients suffer a CFR of up to 14% (refs. ^13,18^); thus, the development of effective drug treatments for these patients is a matter of urgency. Recently, concerns were raised about whether the administration of antihypertensive drugs angiotensin II receptor blockers (ARBs) and angiotensin-converting enzyme inhibitors (ACEIs), inhibitors of the renin–angiotensin–aldosterone system (RAAS), to COVID-19 patients would worsen disease progression through the upregulation of ACE2 expression level, resulting more severe SARS-CoV-2 infection^19^. However, a recent study indicated that RAAS inhibitors do not increase the risk of COVID-19 requiring admission to hospital, including fatal cases and those admitted to intensive care units (ICUs)^20^. In this study, we tested a panel of antihypertensive drugs that are in clinical use and found that the CCBs benidipine HCI and amlodipine besylate have significant antiviral effect in vitro. A retrospective clinical investigation showed that amlodipine besylate was associated with a decreased CFR of COVID-19 patients with hypertension as the only comorbidity. These results provide a valuable reference for selecting drug treatment for COVID-19 patients with hypertension as the underlying comorbidity.

## Results

### CCBs inhibit SARS-CoV-2 infection in vitro

**Fig. 1:**
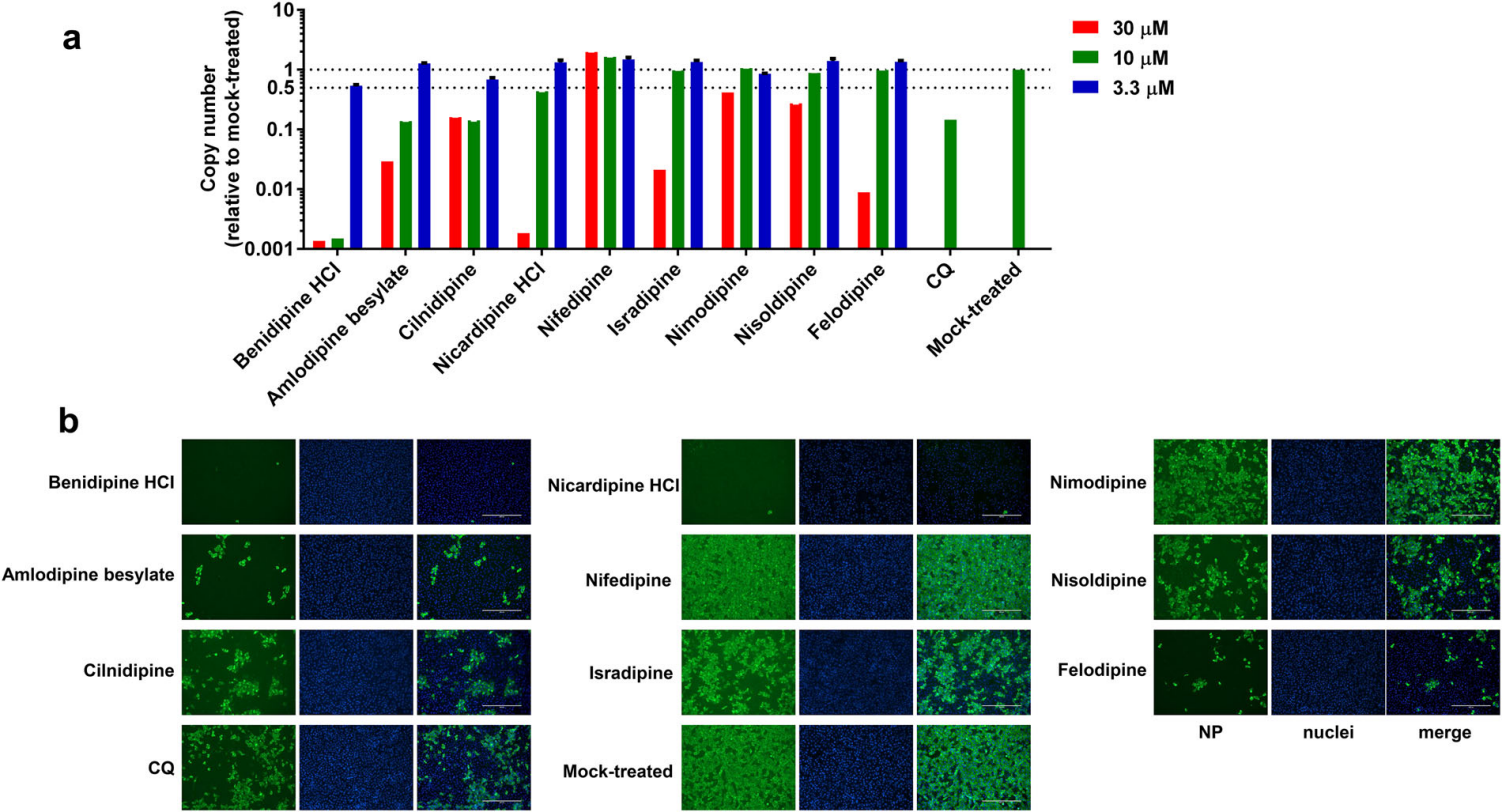
Evaluation of the anti-SARS-CoV-2 activity of a panel of CCBs. Vero E6 cells were treated with the indicated concentrations of compounds and infected with SARS-CoV-2 at an MOI of 0.05, and at 24 h p.i., supernatant was collected and the cells were fixed. Chloroquine (CQ; 5 μM) was used as positive control. **a** Viral RNA copy number in the supernatant was measured with quantitative RT-PCR; **b** the intracellular NP levels in cells treated with 30 μM of the indicated compound were monitored with immunofluorescence. The experiments were performed in triplicates, and the data shown are means ± standard deviation (SD). Bars: 400 μm.

**Fig. 2:**
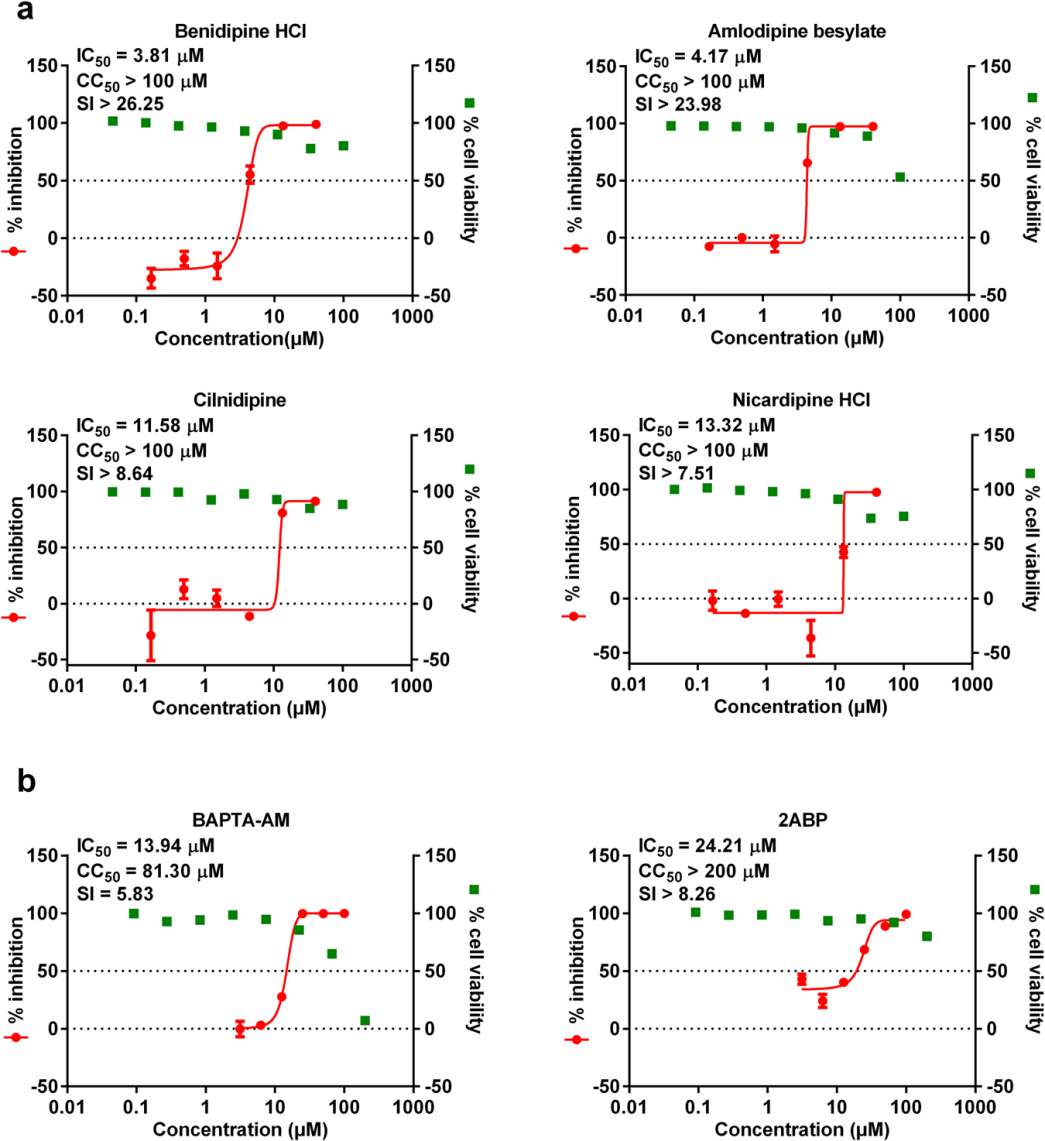
Dose-dependent effects of benidipine HCI, amlodipine besylate, cilnidipine, nicardipine HCl, BAPTA-AM, and 2ABP on SARS-CoV-2 replication. Vero E6 cells were treated with the indicated concentrations of benidipine HCI, amlodipine besylate, clinidipine, nicardipine HCI (**a**), BAPTA-AM or 2ABP (**b**) and were infected with SARS-CoV-2 at an MOI of 0.05, and at 24 h p.i., the supernatant was collected and used to measure the viral copy number using quantitative RT-PCR. Cell viability was measured using a CCK-8 assay. The left *Y*-axis of the graph indicates the mean % inhibition of the virus, while the right *Y*-axis represents mean % cell viability. The experiments were done in triplicates, and the data shown are means ± SD. The half maximal inhibitory concentrations (IC_50_) and half cytotoxic concentration (CC_50_) values were calculated by GraphPad Prism 6.0.

To test whether CCBs can inhibit SARS-CoV-2 replication, Vero E6 cells were treated with a panel of nine clinically approved CCBs, and then infected with SARS-CoV-2 at a multiplicity of infection (MOI) of 0.05. At 24 h post infection (p.i.), the viral RNA copy number in the supernatant was measured using quantitative real-time polymerase chain reaction (RT-PCR; Fig. 1a), and the intracellular level of virus infection was monitored by immunofluorescence with an antibody against virus NP protein (Fig. 1b). We found that all these CCBs can inhibit SARS-CoV-2 replication (Fig. 1 and Supplementary Fig. S1), among which benidipine HCI, amlodipine besylate, cilnidipine, and nicardipine HCI showed more significant inhibition effect (Fig. 1). Experiments with serial concentrations of drug treatment revealed that these four CCBs inhibited SARS-CoV-2 replication in a dose-dependent manner, without causing strong cytotoxic effect (Fig. 2a and Supplementary Fig. S2). The half maximal inhibitory concentrations (IC_50_) of benidipine HCI, amlodipine besylate, cilnidipine, and nicardipine HCI were 3.81, 4.17, 11.58, and 13.32 μM, respectively, and the half cytotoxic concentration (CC_50_) for all four drugs were calculated to be above 100 μM. The drug selective index (SI = CC_50_/IC_50_) of these four CCBs was calculated to be >26.25, >23.98, >8.64, and >7.51, respectively (Fig. 2a). Similar inhibition effects from these four CCBs were also observed on the human hepatocyte cell line Huh7 (Supplementary Fig. S3). The anti-SARS-CoV-2 activity of benidipine HCI and amlodipine besylate was also confirmed by plaque assay (Supplementary Fig. S4).

As CCBs block intracellular calcium influx, we analyzed whether the anti-SARS-CoV-2 effect of CCBs is related to reduced intracellular calcium levels. The intracellular calcium level can be reduced through treatment with calcium chelator 1,2-bis(2-aminophenoxy)ethane-N,N,N′,N′-tetraacetic acid tetrakis(acetoxymethyl ester) (BAPTA-AM) or 2-aminoethyl diphenylborinate (2APB), a membrane-permeable blocker of the inositol 1,4,5-trisphosphate-induced Ca^2+^ release^21^. Vero E6 cells were treated with serial concentrations of BAPTA-AM or 2APB, and then infected with SARS-CoV-2. At 24 h p.i., the viral RNA copy number in the supernatant was measured using qRT-PCR. As shown in Fig. 2b, the addition of BAPTA-AM or 2APB also significantly inhibited virus replication in a concentration-dependent manner, confirming the dependence of SARS-CoV-2 replication on intracellular Ca^2+^ levels, and either blocking of Ca^2+^ flux from medium or ER to cytoplasm can inhibit SARS-CoV-2 replication.

### CCB inhibits viral replication at the post-entry stage

**Fig. 3:**
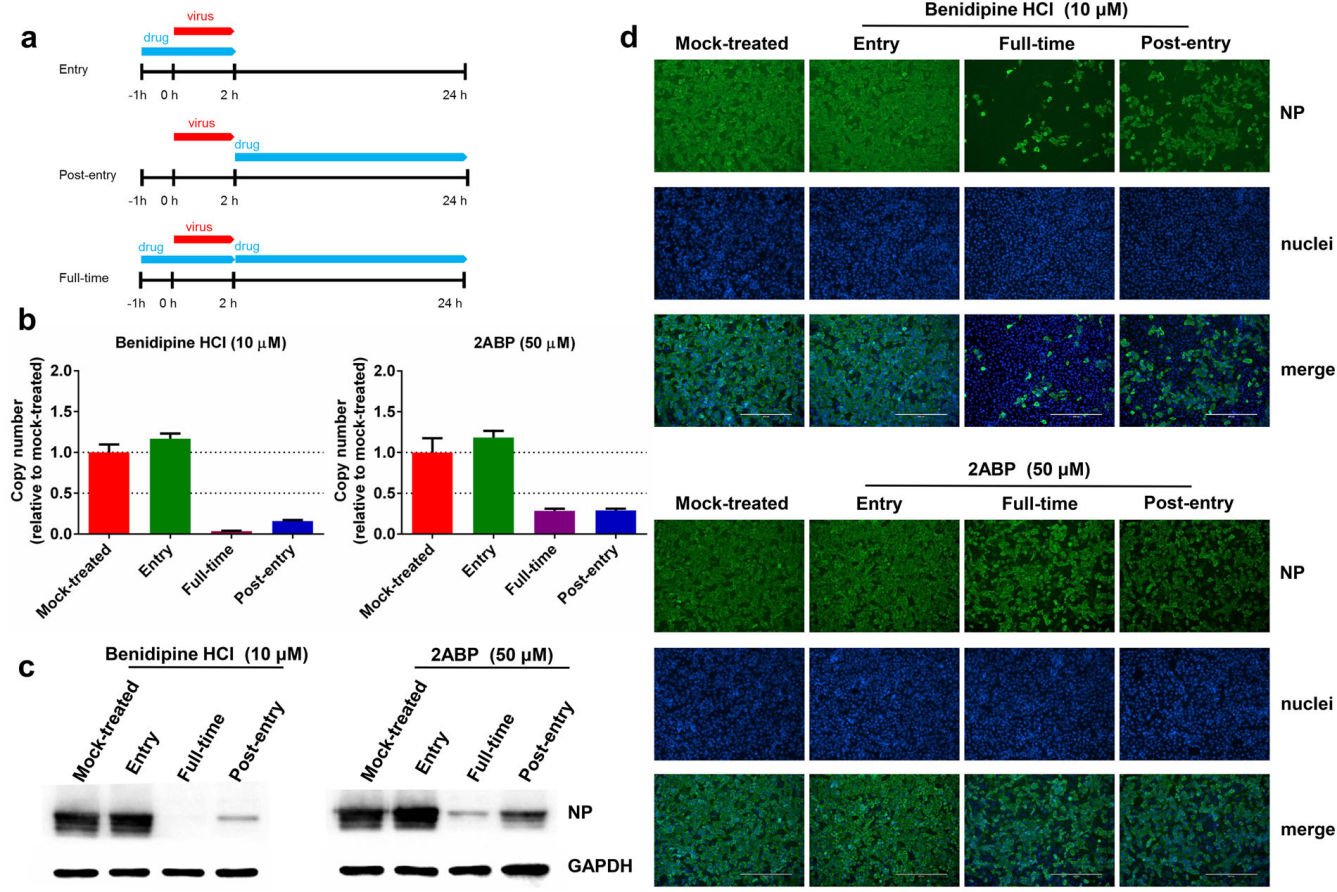
Time-of-addition experiment of benidipine HCI and 2ABP. **a** For “full-time” treatment, Vero E6 cells were pretreated with compounds for 1 h, and then infected with the virus. At 2 h p.i., the supernatant was removed, and the cells were cultured with medium containing the compound until the end of the experiment. For “entry” treatment, Vero E6 cells were pretreated with compounds for 1 h, and then infected with virus. At 2 h p.i., the supernatant was removed, and the cells were cultured with fresh culture medium until the end of the experiment. For “post-entry” experiment, Vero E6 cells were infected with virus, and at 2 h p.i., cells were treated with medium containing the compound until the end of the experiment. For all of these experiments, Vero E6 cells were infected with SARS-CoV-2 at an MOI of 0.05, and the virus copy number in the supernatant was quantified by quantitative RT-PCR (**b**), and NP expression in infected cells was analyzed by western blot (**c**) and immunofluorescence using an NP antibody (**d**) at 24 h p.i. The *Y*-axis of the graph represents mean % inhibition of virus. The experiments were performed in triplicates. Bars: 400 μm.

To define the specific event of virus infection that was inhibited by CCBs, a time-of-addition assay was performed for drug treatment. The CCB benidipine HCI was chosen for further analysis as it has the lowest effective concentration and the highest SI index of the four tested CCBs. Benidipine HCI or 2ABP were added during virus entry, 2-h post virus infection or throughout virus infection (Fig. 3a). The virus production in the supernatant was measured using qRT-PCR and the intracellular NP expression levels were determined by western blot and immunofluorescence analysis, using the NP antibody. As shown in Fig. 3b–d, the addition of drug throughout virus infection or 2-h after virus entry strongly inhibited virus production, while the addition of drug during virus entry did not inhibit virus replication. Notably, compared with drug treatment throughout virus infection, the addition of drugs 2-h after virus entry had slightly lower inhibition efficacy (Fig. 3b, c). Further characterization is needed to determine whether this is due to incomplete blocking by drug treatment due to the rapid onset of virus replication within the first 2 h. Nevertheless, these results indicate that benidipine HCI and 2ABP mainly inhibit virus infection at a stage after virus entry, potentially during virus genome replication/transcription.

### CCBs but not ARBs or ACEIs display inhibitory effect against SARS-CoV-2 replication in vitro

**Fig. 4:**
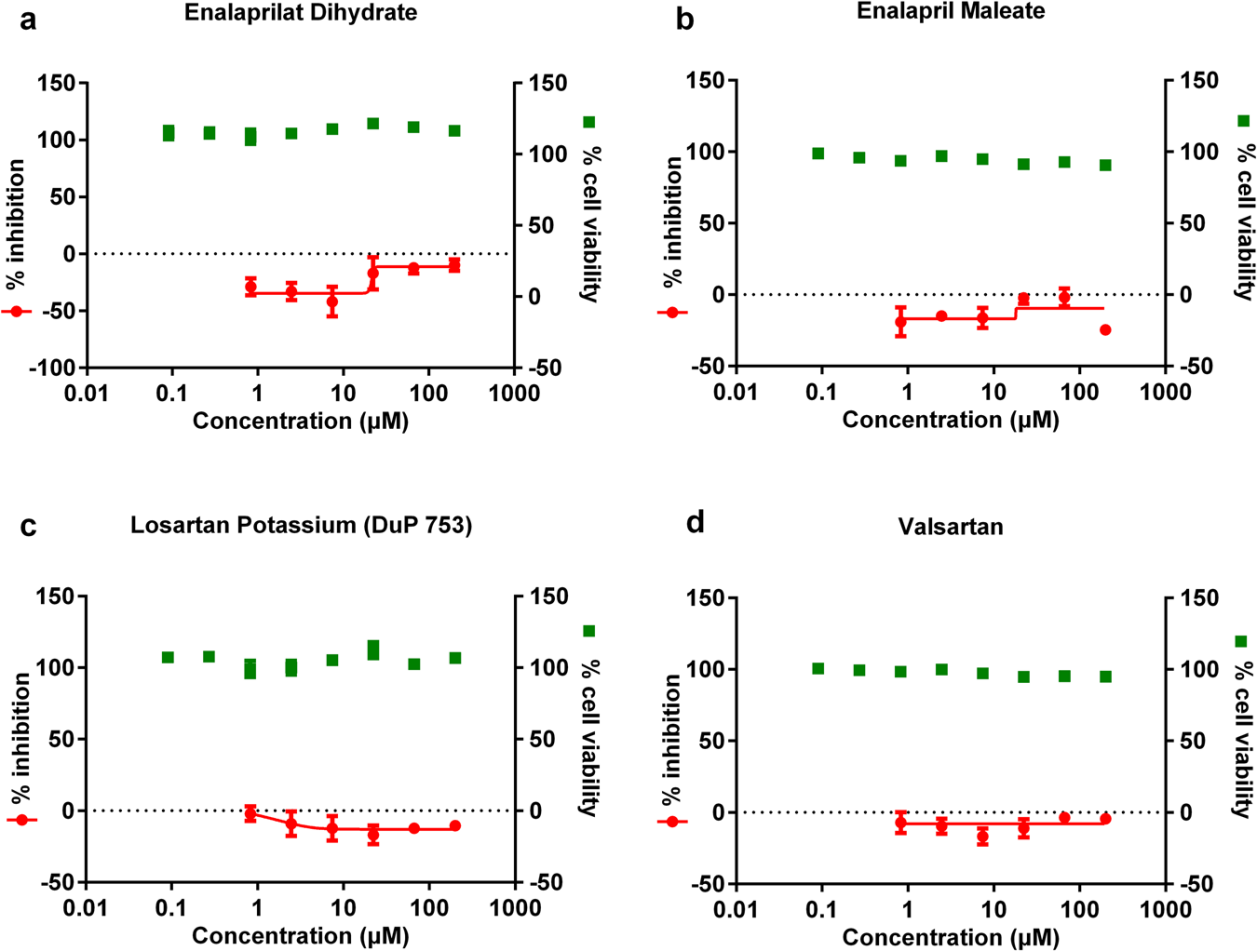
Effect of drug treatment with two ACEIs (enalaprilat dihydrate and enalapril maleate) or two ARBs (losartan potassium and valsartan) on SARS-CoV-2 replication in vitro. Vero E6 cells were treated with the indicated concentrations of enalaprilat dihydrate (**a**), enalapril maleate (**b**), losartan potassium (**c**), or valsartan (**d**), and were infected with SARS-CoV-2 at an MOI of 0.05. At 24 h p.i., the supernatant was collected to measure viral copy number using quantitative RT-PCR. The cell viability was measured using a CCK-8 assay. The left *Y*-axis of the graph indicates the mean % inhibition of the virus, while the right *Y*-axis represents mean % cell viability. The experiments were performed in triplicates, and the data shown are means ± SD.

ARBs, ACEIs, and CCBs represent three major types of antihypertensive drugs that are in clinical use^22^. We next analyzed whether the ARB and ACEI antihypertensive drugs can also inhibit SARS-CoV-2 replication. Representative ARBs (losartan potassium and valsartan) or ACEIs (enalaprilat dehydrate and enalapril maleate), which are widely used in the clinics^22^, were chosen for the evaluation of potential antiviral effects. Vero E6 cells were treated with serial concentrations of drug compounds and were infected with SARS-CoV-2 at an MOI of 0.05. At 24 h p.i., the viral RNA copy number in the supernatant was measured with qRT-PCR, and the cell viability was measured using a CCK-8 assay. As shown in Fig. 4, the selected ARBs or ACEIs did not show any significant inhibition effects, in contrast to the distinct inhibition efficacy of CCBs against SARS-CoV-2. These results suggest that of the three types of antihypertensive drugs, only CCBs have significant anti-SARS-CoV-2 efficacy in vitro.

### Combined application of chloroquine with CCB results in an enhanced anti-SARS-CoV-2 effect

**Fig. 5:**
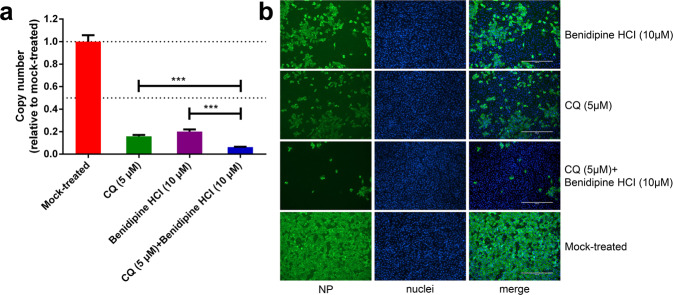
The antiviral activities of chloroquine and/or benidipine HCI against SARS-CoV-2 replication. Vero E6 cells were treated with the indicated concentrations of compounds separately or in combination, and were infected with SARS-CoV-2 at an MOI of 0.05. At 24 h p.i., the supernatant was collected to measure the viral RNA copy number using quantitative RT-PCR (**a**), and the NP expression in infected cells was analyzed by immunofluorescence using an NP antibody (**b**). The experiments were performed in triplicates, and the data shown are means ± SD. Comparison of mean values between two groups was analyzed by the Student’s *t* test. *P < 0.05; **P < 0.01; ***P < 0.001. Bars: 400 μm.

CQ was recently reported to inhibit the entry stage of SARS-CoV-2 (ref. ^5^). Considering that CCB may inhibit SARS-CoV-2 at the post-entry stage, we analyzed whether the combined application of CQ and CCB would lead to a more effective inhibition effect. CQ and CCB were added to the Vero E6 cells either separately or in combination, followed by virus infection with SARS-CoV-2 at an MOI of 0.05. At 24 h p.i., the viral RNA copy number of the supernatant was measured with qRT-PCR, and the intracellular levels of the virus infection were monitored by immunofluorescence using the NP antibody. As shown in Fig. 5, while the separate application of CQ or benidipine HCI resulted in a distinct reduction in virus replication, the combined application of benidipine HCI and CQ further enhanced the anti-SARS-CoV-2 efficacy (*P* < 0.001). We further explored whether the inhibition effect of CQ with CCB is synergistic or additive. Drug interaction was evaluated using the checkerboard assay with serially twofold (0–20 μM) diluted CQ and benidipine HCI in combination. The zero interaction potency model^23^ was used to analyze the interaction of the two compounds using SynergyFinder^24^. Benidipine HCI and CQ in combination yielded a synergy score of −1.922 (Supplementary Fig. S5), indicating that the combination is additive.

### Administration of amlodipine besylate is associated with a decreased case fatality rate in COVID-19 patients with hypertension

In order to evaluate whether CCBs have a therapeutic effect in COVID-19 patients, we retrospectively analyzed the medical records of 225 adult COVID-19 patients with hypertension who had been admitted into the Tongji Hospital from 17 January to 14 February. Of these patients, 119 concurrently had other underlying comorbidities, such as diabetes, chronic obstructive pulmonary disease, cerebral infarction, and 10 were still in the hospital. The remaining 96 patients, who had only hypertension as a comorbidity and who were either discharged from the hospital or were deceased, were included for the final analysis. Among these patients, 19 received amlodipine besylate, 14 received nifedipine, 8 received ARBs/ACEIs, 45 had no drug information, and 10 had no antihypertension drug treatment. No patient was found to be receiving benidipine HCI treatment. All of the patients who did not receive amlodipine besylate were defined as non-amlodipine besylate-treated patients. For amlodipine besylate-treated and non-amlodipine besylate-treated patients, the median (IQR) age was 65 (59–71) and 67 (58.5–72.5) years, the female proportion was 63.2% and 42.9%, and the median (IQR) delay from symptom onset to hospital admission was 10 (7–15) and 10 (7–15.5) days, respectively. None of the three variables showed significant intergroup difference (each *P* > 0.05, Table 1). The frequencies of clinical manifestations that were recorded before or at admission, including fever, cough, feeble, chest distress, shortness of breath, and gastrointestinal symptoms, were comparable between the two groups (all *P* > 0.05, Table 1). All laboratory parameters (blood hemogram and biochemical test) tested at admission were comparable, except that the serum total bilirubin level in non-amlodipine besylate-treated group was higher than that in amlodipine besylate-treated group (*P* = 0.020, Table 1). The commonly prescribed therapies during hospitalization included antibiotics, antiviral agents, traditional Chinese medicines, corticosteroids, and respiratory support, all of the therapies were observed with comparable frequencies between the two groups (all *P* > 0.05, Supplementary Table S1). None of the patients has received a treatment with anti-IL6 therapy (tocilizumab).

**Table 1:** Demographic features, clinical characteristics, and outcomes of COVID-19 patients with hypertensive comorbidity.

	Total (*n* = 96)	Amlodipine (*n* = 19)	Non-amlodipine (*n* = 77)	*P* value
Female	45 (46.9)	12 (63.2)	33 (42.9)	0.112
Age, yr, IQR	66.5 (59–72)	65 (59–71)	67 (58.5–72.5)	0.751
Delay from symptom onset to admission	10 (7–15)	10 (7–15)	10 (7–15.5)	0.949
Systolic blood pressure, mmHg	137 ± 17	137 ± 16	137 ± 18	0.995
Diastolic blood pressure, mmHg	84 ± 11	86 ± 10	83 ± 11	0.203
Symptoms at admission^a^				
Asymptomatic	2 (2.1)	1 (5.3)	1 (1.3)	0.358
Fever	66 (68.8)	10 (52.6)	56 (72.7)	0.091
Cough	59 (61.5)	11 (57.9)	48 (62.3)	0.722
Feeble	32 (33.3)	6 (31.6)	26 (33.8)	0.856
Chest distress	28 (29.2)	3 (15.8)	25 (32.5)	0.152
Shortness of breath	30 (31.3)	4 (21.1)	26 (33.8)	0.409
Anorexia	13 (13.5)	3 (15.8)	10 (13.0)	0.717
Nausea or vomiting	10 (10.4)	1 (5.3)	9 (11.7)	0.681
Diarrhea	16 (16.7)	4 (21.1)	12 (15.6)	0.514
Laboratory test results^b^				
WBC	5.77 (4.82–8.57)	7.32 (5.10–9.33)	5.63 (4.79–7.51)	0.276
PLT	212 (149–286)	270 (199–393)	207 (144–259)	0.081
Lymphocyte	0.82 (0.62–1.03)	0.82 (0.54–0.99)	0.82 (0.64–1.18)	0.764
Monocyte	0.42 (0.30–0.65)	0.43 (0.34–0.71)	0.42 (0.29–0.59)	0.536
Neutrophil	4.34 (3.28–6.51)	6.10 (3.58–7.72)	4.06 (3.21–5.95)	0.290
ALT	29 (20–55)	30 (22–45)	28.5 (20–56)	0.611
AST	33 (21–55)	28 (18–43)	34.5 (21–58)	0.422
ALB	34.0 (30.0–37.2)	34.8 (31.9–40.1)	33.9 (30.0–36.9)	0.057
GLB	33.9 (31.1–37.8)	33.3 (28.1–35.4)	34.2 (31.2–38.5)	0.246
TBIL	9.7 (7.4–13.9)	7.4 (6.1–8.8)	10.4 (8.0–14.3)	0.020
ALP	68 (57–84)	62 (53–70)	68 (57–92)	0.431
GGT	33 (21–69)	29 (23–56)	33 (20–71)	0.573
CK	89 (50–166)	114 (40–183)	87 (50–165)	0.856
LDH	321 (247–451)	293 (247–358)	325 (238–497)	0.101
CA	2.29 (2.20–2.42)	2.22 (2.18–2.35)	2.30 (2.20–2.44)	0.531
UREA	4.2 (3.3–6.5)	5.7 (4.1–6.6)	4.0 (3.2–6.4)	0.836
CR	75 (59–90)	80 (58–99)	73.5 (59–88.5)	0.397
Outcomes				
Admission to ICU	5 (5.2)	0	5 (6.5)	0.579
Invasive ventilation	9 (9.4)	0	9 (11.7)	0.197
Fatal	15 (15.6)	0	15 (19.5)	0.037

^a^Clinical manifestations presenting before or at admission.

^b^Laboratory test results that were obtained at admission.

**Fig. 6:**
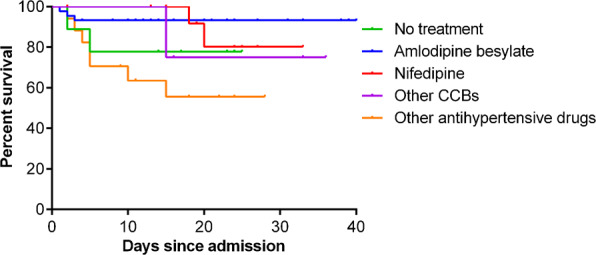
Analysis of amlodipine besylate treatment on probability of survival in COVID-19 patients with hypertension. Treatment effect on probability of survival of amlodipine besylate was compared with patients with other therapy measures. The Kaplan–Meier method was used to analyze time-to-event data.

For the primary outcome of mortality, a beneficial effect in reducing the CFR was observed in patients receiving amlodipine besylate, with the CFR being significantly decreased from 19.5% (15/77) in non-amlodipine besylate-treated group to zero (0/19) in amlodipine besylate-treated group (*P* = 0.037). Kaplan–Meier analysis similarly demonstrated reduced risk of death in amlodipine besylate-treated group, in comparison with non-amlodipine besylate-treated group (*P* = 0.034, log-rank test; Supplementary Fig. S6). Further analysis showed that the CFRs were higher in all other patient groups, with 14.3% (2/14) in patients receiving nifedipine, 25.0% (2/8) in patients receiving ARBs/ACEIs antihypertension drugs, 20.0% (9/45) in patients having no drug information, and 20.0% (2/10) in patients without receiving antihypertension drugs. Potentially due to the limited patient sample size, the difference was only significant between patients having no drug information (*n* = 45) and patients received amlodipine besylate (*n* = 19, *P* = 0.048, Supplementary Table S2), and the treatment effect was also observed in Kaplan–Meier analysis (*P* = 0.034, log-rank test; Fig. 6). The admission to ICU and the use of invasive ventilation were observed in 6.5% (5/77) and 11.7% (9/77) of the non-amlodipine besylate-treated patients, while none of the amlodipine besylate-treated patients needed these treatments, although the intergroup comparison showed no significance (Table 1).

## Discussion

Among COVID-19 patients, the most commonly reported symptoms include cough, fever, and shortness of breath, as well as other major comorbidities, such as hypertension and diabetes^25^, which is associated with endothelium dysfunction. It has been reported that nearly half of COVID-19 patients have comorbidities, with hypertension being the most common comorbidity, followed by diabetes^13^. The occurrence of severe disease was significantly higher in patients with diabetes^26,27^, and hyperglycemia was associated with a higher risk of severe disease^28–30^. Furthermore, compared to normoglycemic patients, administration of tocilizumab failed to attenuate risk of severe outcomes in hyperglycemic patients^28^. Around 13%–30% of COVID-19 patients have hypertension as an underlying comorbidity^13,18,31^, and the CFR in this group of patients is calculated to be over sixfold higher than that in the patients without an underlying comorbidity^31^. ARBs, ACEIs, and CCBs are three major types of antihypertensive drugs that are widely used in the clinics. It has been reported that the ARBs or ACEIs, such as losartan, olmesartan, and lisinopril, lead to higher cardiac ACE2 mRNA levels in animal model^32,33^. As the SARS-CoV-2 virus uses ACE2 as its entry receptor, this raises concerns as to whether administration of these two types of drugs would lead to higher expression level of ACE2, resulting in more severe virus infection^19^. However, clinical studies indicated that ARBs and ACEIs do not increase the risk of COVID-19 requiring admission to hospital^20^. Meanwhile, we found that treatment with ARBs or ACEIs did not show any promotion or inhibition effects on SARS-CoV-2 in vitro. Instead, we showed here that, of the three types of antihypertensive drugs, only CCBs such as, benidipine HCI or amlodipine besylate, presented potent anti-SARS-CoV-2 activity in vitro. Following administration of 8 mg of benidipine HCI or 5 mg of amlodipine besylate or 20 mg of cilnidipine or 60 mg nicardipine hydrochloride in volunteers, the Cmax are ~4 ng/mL for both amlodipine besylate and benidipine HCI, 16.5 ng/mL for cilnidipine, and 58.4 ng/mL for nicardipine hydrochloride, respectively^34^, which is below the in vitro inhibition concentration of SARS-CoV-2 in Vero E6 cells. However, the retrospective clinical investigation on 96 COVID-19 patients with hypertension as the only comorbidity revealed the beneficial effects of amlodipine besylate administration with decreased CFR (0%, *n* = 19). In contrast, the general CFR of this group of patients is 15.6% (*n* = 96). These results together suggest that CCBs, such as amlodipine besylate, may be effective drug options for treating COVID-19 patients who have hypertension as the comorbidity. The benefit of CCBs on COVID-19 patients with hypertension was also observed by other research groups^35–37^. Meanwhile, we cannot ignore the limitation of the small sample size, and loss of information regarding biomarkers of cardiac damage, lung echography, and IL6 level in patients in the current study; therefore, further studies including larger sample size are needed to confirm the efficacy of CCBs in treating COVID-19 patients who have hypertension as the comorbidity.

The therapeutic mechanism of CCBs against COVID-19 still awaits further investigation. Several pathogenic viruses, such as the Zika virus, dengue virus, and H5N1 avian influenza virus, induce intracellular calcium influx to facilitate virus infection^38,39^. The elevation of intracellular calcium levels is associated with pathogenesis mechanisms, including the induction of mitochondrial dysfunction and cell death, which will result in the triggering of strong inflammatory responses^40–42^.

Consistently, CCBs were reported to have anti-inflammatory efficacy through regulating intracellular calcium levels in patients, and to decrease the mortality in septic animal models with excessive inflammatory responses^43,44^. Particularly, amlodipine besylate has been shown to decrease levels of inflammatory markers and oxidative stress compared with the baseline in patients with hypertension^45^. Excessive inflammatory responses are reported to be associated with a fatal COVID-19 outcome^13^. It is possible that, besides inhibiting virus replication, CCBs may also function through alleviating inflammatory responses in the patients to achieve the clinical benefits in a synergistic way with its antiviral efficacy.

CCBs have recently been reported to inhibit the replication of several emerging viruses, including the Ebola virus, Marburg virus^46,47^, Junin virus^16^, and severe fever with thrombocytopenia syndrome virus (SFTSV)^15^. Particularly, CCB treatment was reported to be associated with reduced CFR among SFTS patients^15^. Here, we show that, similar with SFTSV, CCBs inhibit the post-entry events of SARS-CoV-2 replication. Although the exact inhibition mechanism still needs further investigation, it is possible that CCBs block the virus-induced intracellular calcium influx and impair the calcium-dependent cellular pathways that are critical for virus replication. In this way, CCBs may function as a host-oriented drug that inhibits virus replication through regulating the virus-dependent host machinery and the chance for the occurrence of resistant mutants is lower compared with the antiviral drugs that target specific virus constituents^48^. This would be highly valuable for developing drugs against RNA viruses, such as SARS-CoV-2 as these viruses generally have a high mutation rate.

CQ has been shown to efficiently block SARS-CoV-2 entry in vitro and emerging evidences showed that the administration of CQ has beneficial effects for COVID-19 patients in clinics. It has also been reported that the administration of CQ can reduce the overall inflammation in several conditions with little toxicity^6^. Whether CQ also alleviates the excessive inflammatory responses in COVID-19 patients is currently unknown. Nevertheless, the significantly enhanced anti-SARS-CoV-2 efficacy upon combined application of CQ and CCB indicates that the dual administration of these two drugs may achieve a more pronounced therapeutic effect. Several clinical trials are currently ongoing for analyzing the therapeutic effect of CQ in COVID-19 patients. It would be interesting to evaluate any patients that have received a combined drug treatment of CQ and CCBs.

The results from this study suggested that the CCB amlodipine besylate therapy is associated with a decreased CFR of COVID-19 patients with hypertension. COVID-19 patients with several comorbidities besides hypertension may have a more complicated underlying condition, and, therefore, were not included in the current study. Thus, the therapeutic potential may only be applicable to patients with hypertension as the only comorbidity. Evaluation with larger-scale cohort studies or randomized controlled trials would further verify the potential therapeutic effects of the CCBs for this group of COVID-19 patients. In addition, dosing, side-effects, and drug–drug interactions of the CCBs, similar with any drug that is in clinical use or testing, should be rigorously evaluated before the clinical benefits can be more formally concluded.

## Materials and methods

### Cells, virus, and reagents

Vero E6 cell line was obtained from American Type Culture Collection and maintained in minimum Eagle’s medium (Gibco Invitrogen) supplemented with 10% fetal bovine serum (FBS; Gibco Invitrogen) and 1% antibiotic/antimycotic (Gibco Invitrogen), at 37 °C in a humidified 5% CO_2_ incubator. Huh7 cell line was cultured in Dulbecco’s modified Eagle’s medium (DMEM; Gibco Invitrogen) supplemented with 10% FBS and 1% antibiotic/antimycotic (Gibco Invitrogen), at 37 °C in a humidified 5% CO_2_ incubator.

SARS-CoV-2 (nCoV-2019BetaCoV/Wuhan/WIV04/2019) was propagated in Vero E6 cells^2^, and viral titer was determined by 50% tissue culture infective dose (TCID50) as described in our previous study^5^. All the infection experiments were performed in a biosafety level-3 (BSL-3) laboratory.

Benidipine HCI (Selleck Chemicals, no. S2017), amlodipine besylate (Selleck Chemicals, S1813), cilnidipine (Selleck Chemicals, S1293), nicardipine HCl (Selleck Chemicals, S4181), nifedipine (Selleck Chemicals, S1808), isradipine (Selleck Chemicals, S1662), nimodipine (Selleck Chemicals, S1747), nisoldipine (Selleck Chemicals, S1748), felodipine (Selleck Chemicals, S1885), 2APB (Selleck Chemicals, S6657), BAPTA-AM (Selleck Chemicals, S7534), and CQ (Sigma-Aldrich, no.C6628) were purchased from indicated companies.

### Evaluation of the antiviral activities of the test compounds

Vero E6 pre-seeded in 48-well dish (1 × 10^5^ cells/well) were treated with the different concentration of the indicated compounds for 1 h and infected with SARS-CoV-2 at an MOI of 0.05. Two hours later, the virus–drug mixture was removed and cells were cultured with drug containing medium. At 24 h p.i., the cell supernatant was collected and lysed. The viral RNA extraction and qRT-PCR analysis was described in our previous study^5^.

### Evaluation of the cytotoxicity of the test compounds

Vero E6 pre-seeded in 96-well dish (5 × 10^4^ cells/well) were treated with the different concentration of the indicated compounds, and 24 h later, the relative numbers of surviving cells were measured with cell counting kit-8 (GK10001, GLPBIO) according to the manufacturer’s instructions.

### Immunofluorescence microscopy

To detect the intracellular expression level of viral NP, cells were fixed with 4% paraformaldehyde in advance. Fixed cells were permeabilized with 0.5% Triton X-100 and blocked with 5% bovine serum albumin (BSA). Then they were incubated for 2 h with the anti-sera (1:1000 dilution) against the NP of a bat SARS-related CoV as the primary antibody, followed by incubation with Alexa 488-labeled goat anti-rabbit IgG (Abcam, ab150077; 1:500 dilution). The nuclei were stained with DAPI (Sigma-Aldrich, no. D9542). The images were taken by a fluorescence microscopy.

### Western blot analysis

For western blot analysis, proteins were separated by 12% SDS–PAGE and then transferred onto PVDF membranes (Millipore). The membranes were blocked with 5% BSA in TBST (TBS buffer with 0.1% Tween 20) for 1 h at room temperature. After washed with TBST for three times, the membranes were incubated with the anti-NP sera (1:2000 dilution) overnight at 4 °C. After washed with TBST for three times, the membranes were incubated with horseradish peroxidase-conjugated goat anti-rabbit IgG (Proteintech, China; 1:10000 dilution). Protein bands were detected by SuperSignal West Pico Chemiluminescent substrate (Pierce).

### Plaque assay

Vero E6 cells (1 × 10^5^ cells/well) were incubated with supernatant containing SARS-Cov-2. At 1 h p.i., the supernatant was removed, and the cells were incubated under an overlay consisting of DMEM supplemented with 2% FBS and 0.9% CMC (Calbiochem). At 4 days p.i., the overlay was discarded and cells were fixed for 30 min in 4% polyoxymethylene, and stained with crystal violet working solution.

### Clinical investigation

#### Study design and patients

To investigate the clinical effect of amlodipine treatment on COVID-19, we conducted a retrospective clinical investigation on the patients who were admitted to the Tongji Hospital, Union Hospital, which are the major tertiary teaching hospitals in Wuhan, China, and are responsible for the treatments of severe COVID-19 cases. Diagnosis of COVID-19 was made according to the clinical criteria of diagnosis and discharge standards for “Diagnosis and Treatment Scheme of New Coronavirus Infected Pneumonia”. (Released by National Health Commission and State Administration of Traditional Chinese Medicine on March 3, 2020). Briefly, the patients who had epidemiology history, clinical manifestations that mimic COVID-19 were diagnosed after examination of SARS-CoV-2 RNA by RT-PCR and chest computed tomography scanning. Adult confirmed patients were checked for medical records of comorbidities and related therapeutic drugs by a trained research medical staff, and the COVID-19 patients who had hypertension were recruited into the study. Patients, who had other comorbidities, such as coronary heart diseases, cerebral infarction, diabetes, chronic obstructive pulmonary disease, pulmonary tuberculosis, chronic kidney disease, and malignancy, were excluded. The research protocol was approved by the human ethics committee of the hospital in accordance with the medical research regulations of China (TJ-IRB20200102), and oral informed consents were obtained from all patients or patients’ family members.

#### Study inclusions and exclusion criteria

We retrospectively analyzed the medical records of 225 adult COVID-19 patients with hypertension who had been admitted into the Tongji Hospital from 17 January to 14 February. Of these patients, 119 concurrently had other underlying comorbidities, such as diabetes, chronic obstructive pulmonary disease, cerebral infarction, and 10 were still in the hospital. The remaining 96 patients, who had only hypertension as a comorbidity and who were either discharged from the hospital or were deceased, were included for the final analysis.

### Data collection

Data about demography, clinical manifestations, and laboratory testing results were retrospectively collected by reviewing medical records and entered into standardized database. Medication use during hospitalization, including information on antihypertensive drugs (i.e., CCBs, ARBs, and diuretics) was also recorded. Serial throat swabs were collected for the testing of HCoV-19 RNA with the use of RT-PCR during the patients’ hospitalization.

### Study outcomes

The primary outcome was all-cause mortality. Secondary outcomes included the admission to ICU and the use of invasive ventilation. The data on fatal outcome, admission to ICU, and the use of invasive ventilation, were retrieved from medical records.

### Statistical analysis

For in vitro study, the Student’s *t* test was used for comparisons of continuous variables between two groups. For clinical study, continuous variables were summarized as means and standard deviations or as medians and IQR. Student’s *t* test or nonparametric test (Mann–Whitney test) was used as appropriate for comparisons of continuous variables between two groups, and ANOVA test or nonparametric test was used as appropriate for comparisons of continuous variables among multiple groups. Categorical variables were summarized as frequencies and proportions, and were analyzed by Chi-square test or Fisher’s exact test as appropriate. We used the Kaplan–Meier method and the log-rank test to analyze time-to-event data for treatment effect analysis. We calculated HRs and 95% CI by using Cox regression models. A two-sided *P* value of < 0.05 was considered to be statistically significant. All statistical analyses were performed using SPSS software, version 19.0.

## Supplementary information

Supplementary information

## Supplementary information

**Supplementary Information** accompanies the paper at (10.1038/s41421-020-00235-0).
