# Author Correction: Calcium channel blocker amlodipine besylate therapy is associated with reduced case fatality rate of COVID-19 patients with hypertension

**DOI:** 10.1038/s41421-021-00267-0

**Published:** 2021-05-03

**Authors:** Lei-Ke Zhang, Yuan Sun, Haolong Zeng, Qingxing Wang, Xiaming Jiang, Wei-Juan Shang, Yan Wu, Shufen Li, Yu-Lan Zhang, Zhao-Nian Hao, Hongbo Chen, Runming Jin, Wei Liu, Hao Li, Ke Peng, Gengfu Xiao

**Affiliations:** 1 State Key Laboratory of Virology, Wuhan Institute of Virology, Center for Biosafety Mega-Science, Chinese Academy of Sciences, Wuhan, Hubei 430071 China; 2 University of the Chinese Academy of Sciences, Beijing, 100049 China; 3 Department of Laboratory Medicine, Tongji Hospital, Tongji Medical College, Huazhong University of Science and Technology, Wuhan, Hubei 430030 China; 4 Tongji Medical College, Huazhong University of Science and Technology, Wuhan, Hubei 430022 China; 5 Department of Pediatrics, Union Hospital, Tongji Medical College, Huazhong University of Science and Technology, Wuhan, Hubei 430022 China; 6 Beijing Institute of Microbiology and Epidemiology, State Key Laboratory of Pathogen and Biosecurity, Beijing, 100071 China

**Keywords:** Mechanisms of disease, Cell biology

## Abstract

A Correction to this paper has been published: https://doi.org/10.1038/s41421-021-00267-0

Correction to: *Cell Discovery* (2020) 6:96

10.1038/s41421-020-00235-0 Published online 22 December 2020

We apologize for the mistake of the author affiliation information. In the initial published version of this article^1^, there was an omission in author affiliations. “University of the Chinese Academy of Sciences, Beijing 100049, China” needs to be added to the affiliation of Yuan Sun, Qingxing Wang, Xiaming Jiang and Gengfu Xiao as the author’s second affiliation. The correct information is as follows. This correction does not affect the description of the results or the conclusion of this work. Lei-Ke Zhang^1^, Yuan Sun^1,2^, Haolong Zeng^3^, Qingxing Wang^1,2^, Xiaming Jiang^1,2^, Wei-Juan Shang^1^, Yan Wu^1^, Shufen Li^1^, Yu-Lan Zhang^1^, Zhao-Nian Hao^4^, Hongbo Chen^5^, Runming Jin^5^, Wei Liu^6^, Hao Li^6^, Ke Peng^1^, Gengfu Xiao^1,2^. ^1^State Key Laboratory of Virology, Wuhan Institute of Virology, Center for Biosafety Mega-Science, Chinese Academy of Sciences, Wuhan, Hubei 430071, China. ^2^University of the Chinese Academy of Sciences, Beijing 100049, China. ^3^Department of Laboratory Medicine, Tongji Hospital, Tongji Medical College, Huazhong University of Science and Technology, Wuhan, Hubei 430030, China. ^4^Tongji Medical College, Huazhong University of Science and Technology, Wuhan, Hubei 430022, China. ^5^Department of Pediatrics, Union Hospital, Tongji Medical College, Huazhong University of Science and Technology, Wuhan, Hubei 430022, China. ^6^Beijing Institute of Microbiology and Epidemiology, State Key Laboratory of Pathogen and Biosecurity, Beijing 100071, China
