# Supplementary material for: Calcium channel blocker amlodipine besylate therapy is associated with reduced case fatality rate of COVID-19 patients with hypertension
Source: Cell Discov. 2020 Dec 22;6:96. doi: 10.1038/s41421-020-00235-0 (PMC7752915; doi:10.1038/s41421-020-00235-0)
Supplement: Supplementary file 1 — Supplementary information [file 41421_2020_235_MOESM1_ESM.pdf]

Supplementary Fig. S1. Evaluation of the anti-SARS-CoV-2 activity of nifedipine, isradipine, nimodipine, nisoldipine and felodipine. Vero E6 cells were treated with the indicated concentrations of compounds and infected with SARS-CoV-2 at an MOI of 0.05, and at 24 hours p.i., supernatant was collected. Viral RNA copy number in the supernatant was measured with quantitative RT-PCR. The experiments were performed in triplicates, and the data shown are means ± standard deviation (SD).

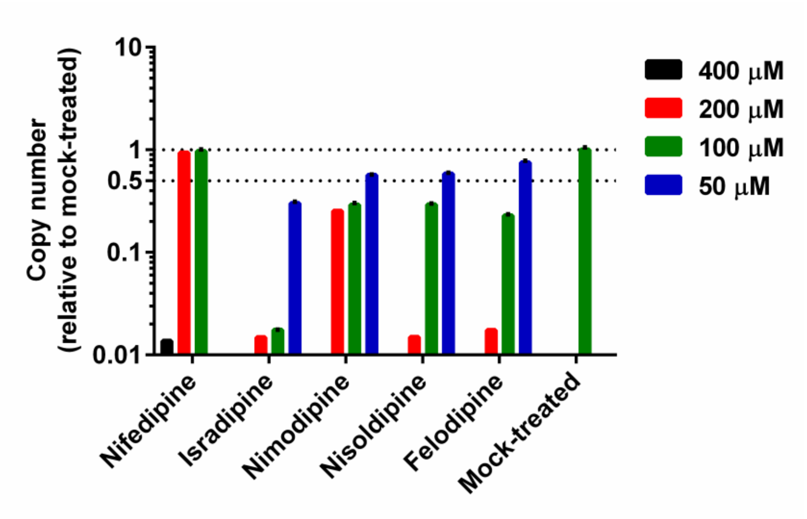

Supplementary Fig. S2. Dose dependent effects of benidipine HCI, amlodipine besylate, cilnidipine, and nicardipine HCl on SARS-CoV-2 replication with an MOI of 0.5. Vero E6 cells were treated with the indicated concentrations of compounds and were infected with SARS-CoV-2 at an MOI of 0.5, and at 24 hours p.i., the supernatant was collected. Viral copy number in the supernatant was measured using quantitative RT-PCR and normalized with mock-treated control. The Y-axis represents mean % copy number. The experiments were done in triplicates, and the data shown are means ± SD.

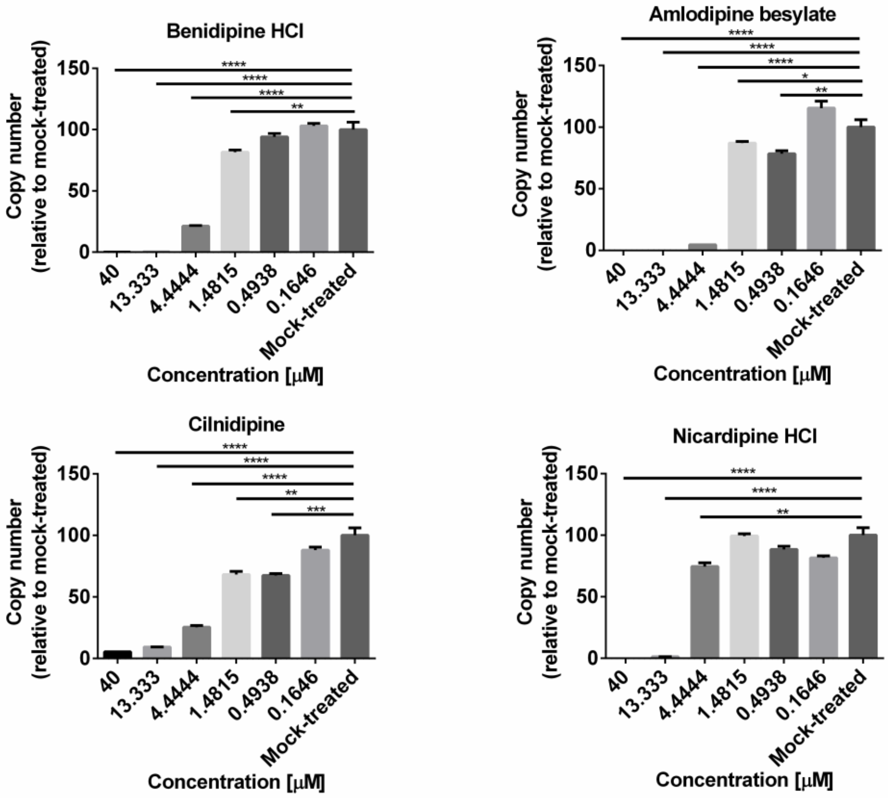

Supplementary Fig. S3. Dose dependent effects of benidipine HCI, amlodipine besylate, cilnidipine, and nicardipine HCl on SARS-CoV-2 replication in Huh7

(**a**) Huh7 cells were treated with the indicated concentrations of compounds and were infected with SARS-CoV-2 at an MOI of 0.05. At 24 hours p.i., the supernatant was collected. Viral copy number in the supernatant was measured using quantitative RT-PCR and normalized with mock-treated control. The Y-axis represents mean % copy number. The experiments were performed in triplicates, and the data shown are means ± SD. (**b**) Huh7 cells were treated with the indicated concentrations of compounds, and 24 hours later, the cell viability was measured using a CCK-8 assay. The Y-axis represents mean % cell viability. The experiments were performed in triplicates, and the data shown are means ± SD. (**c**) Huh7 cells were treated with 10 μM of indicated compounds and infected with SARS-CoV-2 at an MOI of 0.05, and at 24 hours p.i., cells were fixed. Intracellular NP level was monitored with immunofluorescence. Bars: 400 μm. Comparison of mean values between two groups was analyzed by student's t test. *p < 0.05, **p < 0.01, ***p < 0.001, ****p < 0.001.

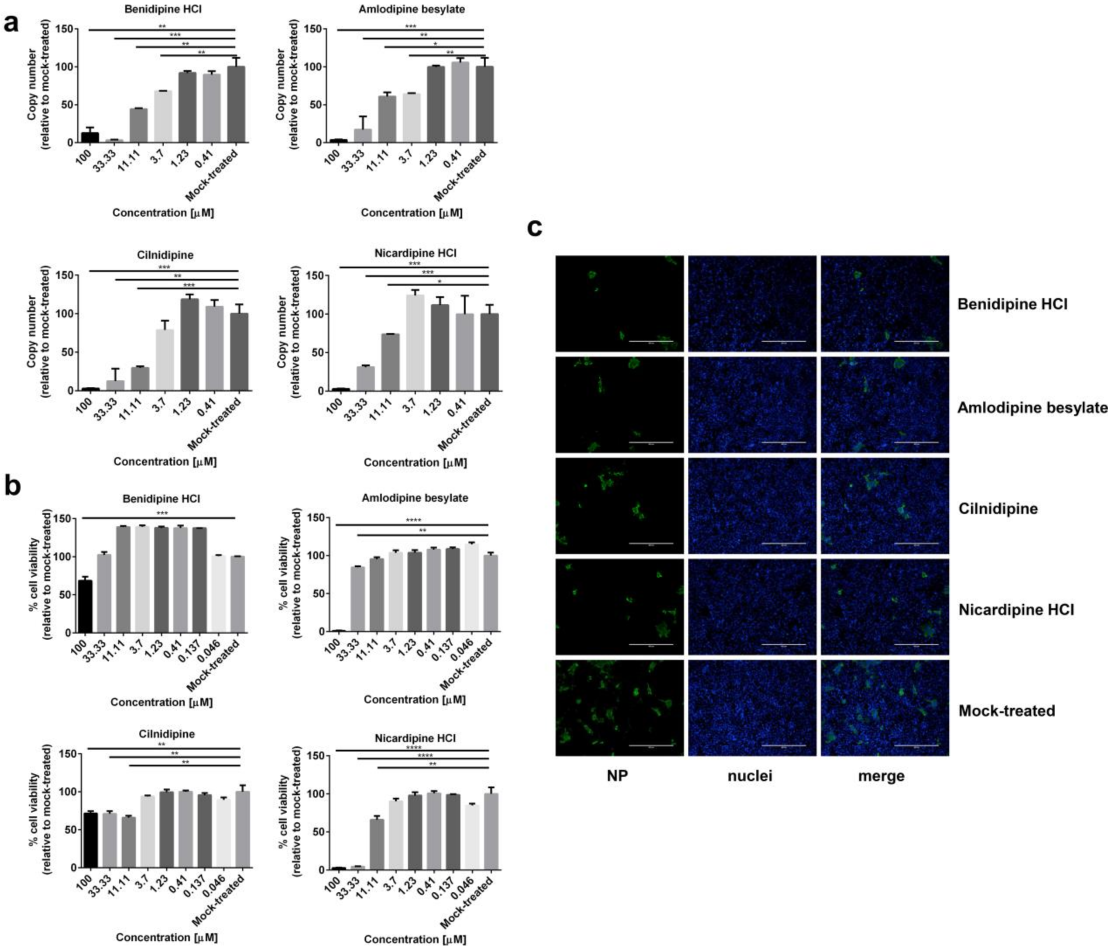
a
Benidipine HCl
Amlodipine besylate
Cilnidipine
Nicardipine HCl
Copy number (relative to mock-treated)
100
33.33
11.11
3.7
1.23
0.41
Mock-treated
Concentration [µM]
b
% cell viability (relative to mock-treated)
0.137
0.046
c
Benidipine HCl
Amlodipine besylate
Cilnidipine
Nicardipine HCl
Mock-treated
NP
nuclei
merge

Supplementary Fig. S4. Dose dependent effects of benidipine HCI, amlodipine besylate on SARS-CoV-2 replication using plaque assay. Vero E6 cells were treated with the indicated concentrations of compounds and were infected with SARS-CoV-2 at an MOI of 0.05, and at 24 hours p.i., the supernatant was collected. Viral titer in the supernatant was measured using plaque assay and normalized with mock-treated control. The Y-axis represents mean % viral titer. The experiments were done in triplicates, and the data shown are means ± SD.

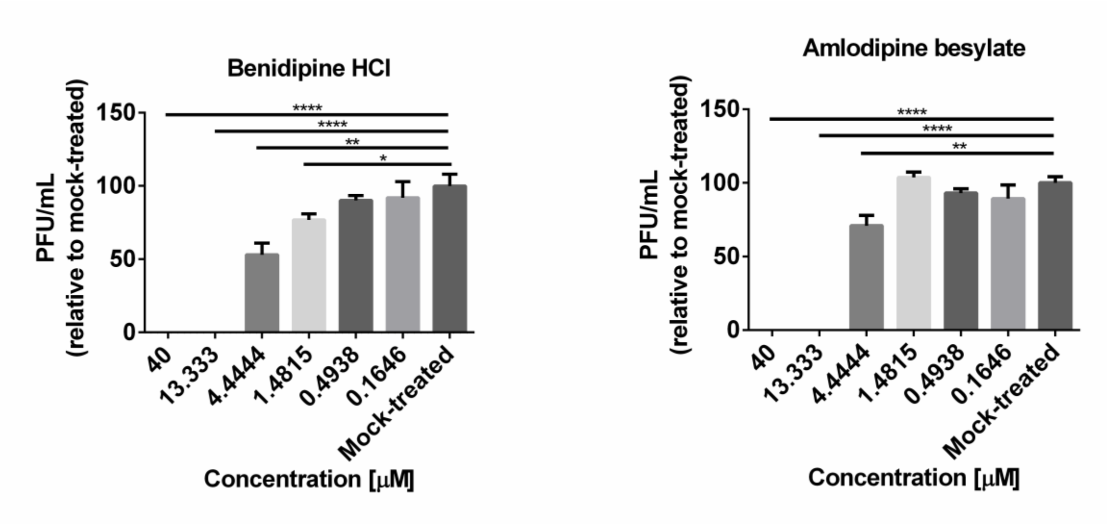

Supplementary Fig. S5. Combinational effect of chloroquine (CQ) and benidipine HCI against SARS-CoV-2 in vitro. (**a**) Dose response matrix of serially 2-fold diluted (0–20 μM) CQ and benidipine HCI in Vero E6 cells. Vero E6 cells were treated with the indicated concentrations of compounds separately or in combination, and were infected with SARS-CoV-2. At 24 hours p.i., cells were fixed and the intracellular NP levels in cells were monitored with immunofluorescence. The percentage of viral inhibition was calculated based on intracellular NP level and normalized to mock-treated control (set as 100%). (**b**) The three-dimensional interaction landscapes of CQ and benidipine HCI were generated by SynergyFinder and synergy score was calculated. Synergy score: Less than -10: the interaction between two drugs is likely to be antagonistic; From -10 to 10: the interaction between two drugs is likely to be additive; Larger than 10: the interaction between two drugs is likely to be synergistic. Red color indicates synergy while the green color indicates antagonism of the two drugs.

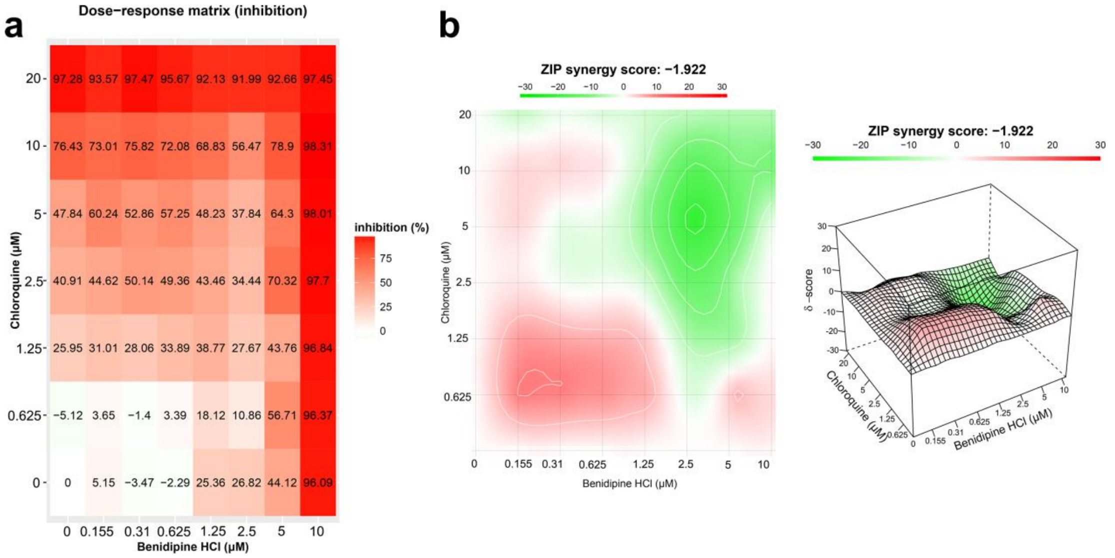

Supplementary Fig. S6. Analysis of amlodipine besylate treatment on probability of survival in COVID-19 patients with hypertension.

Treatment effect on probability of survival of amlodipine besylate was compared with non-amlodipine besylate treated patients. The Kaplan-Meier method was used to analyse time-to-event data.

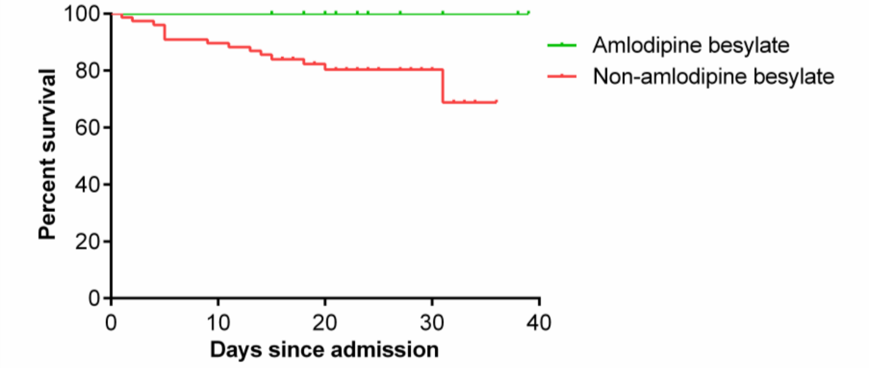

Supplementary Table S1. Therapies administration during hospitalization in COVID-19 patients with hypertensive comorbidity.

	Total (n=96)	Amlodipine (n=19)	Non-amlodipine (n=77)	P
Antibiotics	96 (100)	19 (100)	77 (100)	1.000
Antiviral agents	96 (100)	19 (100)	77 (100)	1.000
Traditional Chinese medicines	77 (80.2)	17 (89.5)	60 (77.9)	0.347
Corticosteroids	46 (47.9)	8 (42.1)	38 (49.4)	0.571
Respiratory support	93 (96.9)	17 (89.5)	76 (98.7)	0.099

Supplementary Table S2. Case fatality rate in COVID-19 patients with hypertension comorbidity

	Total	Survival, n (%)	Fatal, n (%)	P value*
Treatment regimen				
Amlodipine besylate	19	19 (100)	0	
Nifedipine	14	12 (85.7)	2 (14.3)	0.172
ARBs/ACEIs	8	6 (75.0)	2 (25.0)	0.080
Unknown	45	36 (80.0)	9 (20.0)	**0.048**
No treatment	10	6 (80.0)	2 (20.0)	0.111

*The case fatality rates of the patients treated with nifedipine or ARBs/ACEIs, the patients with unknown information on antihypertensive agents, and the patients without antihypertensive treatment, were respectively compared with that of the patients treated with amlodipine besylate. The Chi-square test or Fisher's exact test was performed as appropriate.
